# Regulatory versus coding signatures of natural selection in a candidate gene involved in the adaptive divergence of whitefish species pairs (*Coregonus* spp.)

**DOI:** 10.1002/ece3.52

**Published:** 2012-01

**Authors:** Julie Jeukens, Louis Bernatchez

**Affiliations:** Institut de biologie intégrative et des systèmes (IBIS), Québec-Océan, 1030 av. de la médecine, Université LavalQuébec, QC, G1V 0A6, Canada

**Keywords:** Adaptive divergence, *Coregonus*, gene expression, natural selection, regulatory evolution, speciation

## Abstract

While gene expression divergence is known to be involved in adaptive phenotypic divergence and speciation, the relative importance of regulatory and structural evolution of genes is poorly understood. A recent next-generation sequencing experiment allowed identifying candidate genes potentially involved in the ongoing speciation of sympatric dwarf and normal lake whitefish (*Coregonus clupeaformis*), such as cytosolic malate dehydrogenase (*MDH1*), which showed both significant expression and sequence divergence. The main goal of this study was to investigate into more details the signatures of natural selection in the regulatory and coding sequences of *MDH1* in lake whitefish and test for parallelism of these signatures with other coregonine species. Sequencing of the two regions in 118 fish from four sympatric pairs of whitefish and two cisco species revealed a total of 35 single nucleotide polymorphisms (SNPs), with more genetic diversity in European compared to North American coregonine species. While the coding region was found to be under purifying selection, an SNP in the proximal promoter exhibited significant allele frequency divergence in a parallel manner among independent sympatric pairs of North American lake whitefish and European whitefish (*C. lavaretus*). According to transcription factor binding simulation for 22 regulatory haplotypes of *MDH1*, putative binding profiles were fairly conserved among species, except for the region around this SNP. Moreover, we found evidence for the role of this SNP in the regulation of *MDH1* expression level. Overall, these results provide further evidence for the role of natural selection in gene regulation evolution among whitefish species pairs and suggest its possible link with patterns of phenotypic diversity observed in coregonine species.

## Introduction

Over the last decade, evolutionary and ecological functional genomics has tackled the identification of molecular mechanisms responsible for ecological success and evolutionary fitness in natural populations (Feder and Mitchell-Olds 2003). When aiming at an integrated understanding of all levels of biological organization from DNA to populations, it is necessary to isolate genes of interest, or candidate genes. Recent high-throughput technologies applied to population genomics (Stinchcombe and Hoekstra 2008), transcriptomics (Oleksiak et al. 2002), and proteomics (Biron et al. 2006) have considerably facilitated this first step toward a better understanding of adaptive evolutionary change.

**Figure 1:**
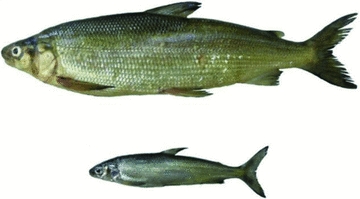
Normal and dwarf lake whitefish (*Coregonus clupeaformis*). Normal whitefish (top) commonly exceeds 40 cm in length and 1000 g in weight while dwarf whitefish (bottom) rarely exceeds 20 cm and 100 g.

Lake whitefish (*Coregonus clupeaformis*, Fig. 1) is one of the most investigated nonclassical models in evolutionary and ecological functional genomics studies. It comprises multiple independently evolved pairs of sympatric forms engaged in a process of ecological speciation. Despite its recent postglacial origin (15,000 YBP, Pigeon et al. 1997), the limnetic dwarf whitefish strikingly differs from the benthic normal whitefish in morphology, but more so in life-history traits, metabolism, and behavior. Transcriptome-wide analyses of gene expression have led to the identification of about 500 candidate genes potentially implicated in the adaptive divergence of dwarf and normal whitefish (reviewed in Bernatchez et al. 2010). Namely, a study of gene transcription in liver tissue using cDNA microarrays revealed parallelism in patterns of gene expression divergence between sympatric forms across controlled and natural environments, thus providing evidence for the role of natural selection in gene regulation evolution between dwarf and normal whitefish (St-Cyr et al. 2008). These results were consistent with the observed trade-off in life-history traits among whitefish species pairs, wherein dwarfs have a higher metabolic rate, necessary for increased foraging and predator avoidance in the limnetic niche, while normal whitefish allocate a much larger fraction of their energy budget to growth (Trudel et al. 2001). A subsequent study focusing on the expression analysis of some of these candidate genes by means of RT-PCR revealed that parallelism in transcription profiles also extended to comparisons between North American and European whitefish (*C. lavaretus*) species pairs (Jeukens et al. 2009).

A recent next-generation sequencing experiment allowed whole liver transcriptome sequencing and efficient single nucleotide polymorphism (SNP) discovery, hence providing a much more comprehensive understanding of transcriptomic divergence between dwarf and normal whitefish (Jeukens et al. 2010; Renaut et al. 2010). Not only did it confirm the results of the aforementioned microarray experiment, but it also demonstrated a decoupling of gene expression and coding sequence divergence (Jeukens et al. 2010). The relative importance of regulatory and structural evolution of genes is not fully understood (Hoekstra and Coyne 2007), yet it seems that these two evolutionary modes did not generally act upon the same genes in whitefish. However, some of them, such as cytosolic malate dehydrogenase, showed significant divergence at both the expression (St-Cyr et al. 2008; Jeukens et al. 2010) and the sequence levels (Renaut et al. 2010), making such candidate genes particularly relevant for further investigation.

Cytosolic malate dehydrogenase (MDH1) catalyses the interconversion of malate and oxaloacetate, the latter being a substrate of gluconeogenesis (Minárik et al. 2002). MDH1 is also involved in the citric acid cycle as it produces malate that is then imported into the mitochondrion through the malate–aspartate shuttle and transformed by the mitochondrial malate dehydrogenase (MDH2, Musrati et al. 1998). MDH1 is a homodimer that forms from two subunits. In contrast to birds and mammals, fish and amphibians have two different subunits, A and B, encoded by two unlinked genes that have undergone limited divergence (Bailey et al. 1969; Bailey et al. 1970; Wheat et al. 1972). These two subunits exhibit tissue specificity such that the A subunit predominates in liver and brain, whereas skeletal muscle contains the B subunit (Bailey et al. 1970). Salmonid fishes have pseudotetraploid genomes due to recent whole genome duplication (Allendorf and Thorgaard 1984), hence they possess four different subunits, A, A’, B, and B’, encoded by two paralogous gene copies for each subunit type (Bailey et al. 1970; Allendorf et al. 1977).

The candidate gene approach has received little attention in studies of whitefish adaptive divergence (e.g., Jeukens et al. 2009), as most of the functional genomics research to date has focused on genome and transcriptome-wide strategies. Now that so many candidate genes have been identified, a possible second step would be to perform an in-depth analysis of regulatory and coding sequence evolution in order to gain insight into the mechanisms and relative importance of these two evolutionary modes. The sequence information that is needed for this type of study is available for very few candidate genes in whitefish, as recent BAC library construction, screening, and clone sequencing for five candidate targets represents the first genomic DNA sequencing effort for this species (Jeukens et al. 2011).

This study focuses on the identification of signatures of selection in the regulatory and coding sequences of cytosolic malate dehydrogenase, which exhibits both expression and coding sequence divergence between dwarf and normal whitefish in addition to being potentially implicated in adaptive metabolic evolution among whitefish species pairs. We also extended the study to test for the occurrence of parallelism in genetic variation at this gene between North American and European whitefish species pairs, which represent natural replicates of intralacustrine evolution of a limnetic whitefish. Two more distantly related coregonine species were also included in order to gain insight into the evolutionary history of MDH1 in the subfamily Coregoninae.

## Materials and Methods

### Samples

Samples used in this study were those described and used for the analysis of gene expression by Jeukens et al. (2009). Briefly, samples from 10 coregonine populations, including four independently evolved sympatric pairs, were used: North American lake whitefish (*C. clupeaformis*) from Cliff Lake (*N*_normal_ = 10, *N*_dwarf_ = 12) and Indian Pond (*N*_normal_ = 12, *N*_dwarf_ = 10, St John River drainage, Maine, USA); European whitefish (*C. lavaretus*) from the Pasvik river catchment (*N*_benthic_ = 13, *N*_limnetic_ = 9, Norway) and lake Zurich (*N*_benthic_ = 11, *N*_limnetic_ = 15, Switzerland); lake cisco (*C. artedi*) from Lac des Trente-et-un-Milles (*N* = 14, Gatineau region, Quebec, Canada); and vendace (*C. albula*) from the Pasvik river catchment (*N* = 12, Norway), for a total of 118 fish. Lake cisco and vendace are two specialized limnetic coregonine species, and previous studies showed that transcription profiles of dwarf whitefish for several genes involved in muscle contraction and energy metabolism have converged to match that of cisco (Derome and Bernatchez 2006; Jeukens et al. 2009). DNA was extracted from liver tissue with a salt extraction method (Aljanabi and Martinez 1997). cDNA samples for these fish were already available and described by Jeukens et al. (2009).

### Primer design

BAC library construction and screening led to full-length assembly of the *MDH1* gene, that is, the complete coding sequence (exons + introns = 6,464 bp) as well as 18,316-bp upstream of the start codon and 1,882-bp downstream of the stop codon (Genbank accession HQ287747, Jeukens et al. 2011). With the goal of obtaining amplicons that could be readily sequenced in both directions for the regulatory and coding sequences of this gene (∼1 kb, see Kohn et al. 2008), two sets of primers were designed: (1) forward 5′-AAATGCGGTGTGCTGTAATGTAGGT-3′ and reverse 5′-AGCTAACACTTTCGATGCATCATTC-3′ (used on genomic DNA, 826-bp amplicon, positions 17,570 to 18,395 of HQ287747); (2) forward 5′- CCTTCTGTTTAGTCCTAGCGGGAAA-3′ and reverse 5′- CAGCGTACACACCCATAGACATGAA-3′ (used on cDNA, 889-bp amplicon, positions 18,249 to 24,126 of HQ287747, without introns). Using cDNA instead of genomic DNA for the coding region allowed us to study most of the complete coding sequence (exons 1–8 out of 9) while avoiding nonspecific amplification due to pseudogenes.

### PCR and sequencing

PCR reactions were carried out in 20-µl volumes (0.5 unit HotStar*Taq* DNA polymerase and 1× PCR buffer [Qiagen, Hilden, Germany], 0.5 µM of each primer) with the following conditions: 15-min activation at 95°C followed by 35 cycles of 45 sec at 95°C, 30 sec at 58°C, and 1 min at 72°C, ending with 5 min at 72 °C. PCR products were then purified with ExoSAP-IT (GE Healthcare, Baie d’Urfe, Canada) and sequenced.

### Sequence processing

Individual inspection and trimming of sequences was performed with BioEdit 7.0.5.2 (Hall 1999). All SNPs were tested for Hardy–Weinberg equilibrium (HWE) within each population using a chi-squared test. Finally, as each sequence was a combination of two alleles, haplotypes were reconstructed with PHASE v.2.1.1 (Stephens et al. 2001). This software implements a Bayesian statistical method for reconstructing haplotypes from population genotype data. PHASE also provides a confidence probability associated with each haplotype combination.

### Sequence annotation

Conserved features of the MDH1 protein, that is, malate binding, nicotinamide adenine dinucleotide (NAD) binding, and dimer interface, were retrieved from NCBI's Conserved domains cd01336. The regulatory sequence was first submitted to various databases: UTRsite (regulatory motifs of the untranslated regions, Mignone et al. 2005), GPMiner (TATA-box, http://gpminer.mbc.nctu.edu.tw/index.php), CpG islands (The Sequence Manipulation Suite, Stothard 2000), and JASPAR CORE Vertebrata (transcription factor binding profile database, Bryne et al. 2008). Because the identification of transcription factor binding sites (TFBSs) is burdened with false positives, the regulatory sequence was also submitted to Sunflower (reference mode), a program that simulates competitive binding of transcription factors based on the JASPAR database in order to associate posterior binding probabilities to putative TFBSs (Hoffman and Birney 2010). Then, in order to perform phylogenetic footprinting, used to circumvent the problem of false positives by identifying TFBSs in conserved regions among species, orthologous sequences were identified in other fish species using the Ensembl genome browser (*Danio rerio*, *Takifugu rubripes*, and *Gasterosteus aculeatus*) and the cGRASP BLAST server (*Salmo salar*, http://web.uvic.ca/grasp/). Two different tools were used for phylogenetic footprinting: ConSite (Sandelin et al. 2004), which compares two orthologous sequences, and the MEME suite (Bailey et al. 2009), which can be used for multiple orthologous sequences in a single analysis.

### General sequence analyses

For the following sequence analyses, HYPHY (Kosakovsky Pond et al. 2005) and its online server Datamonkey (Kosakovsky Pond and Frost 2005a) were used, unless otherwise stated. For this section, all data manipulations were performed for both the coding and noncoding regions.

DNA sequence evolution can be described by various Markov models that differ in terms of the parameters used to define nucleotide replacement rates. These substitution models can be combined with a sequence alignment and its phylogenetic tree to construct a likelihood function. We thus conducted sequence evolution model fitting for our sequence data. Following selection of the most likely substitution model, detection of recombination breakpoints was carried out (GARD, Datamonkey), as recombination can mislead phylogenetic analysis. Phylogenetic reconstruction by Neighbor-Joining based on maximum likelihood estimates (MLE) was performed (NeighborJoining.bf, HYPHY), and the resulting tree was used for sequence evolution model fitting through creation and optimization of a likelihood function (graphical user interface, HYPHY). Different regions of a sequence can be associated with different trees and substitution models, while being part of the same likelihood function. Once MLE for model parameters are available, they can be used for hypothesis testing through likelihood ratio tests (LRTs), for instance, to determine whether substitutions rates are equal between two regions of a sequence. In fact, LRTs are used to compare a given model (alternative hypothesis) with a constrained version of itself (null hypothesis) using the statistic 2(logL_alternative_–logL_null_). A *P*-value is then computed based on the asymptotic chi-squared distribution.

Divergent selection can be inferred in cases where *F*_ST_ values significantly exceed the range of values of polymorphic sites across the genome under neutral expectation (Beaumont and Balding 2004). Thus, adaptive divergence between dwarf and normal whitefish was tested by computing *F*_ST_ estimates based on pairwise genetic distances (Hudson et al. 1992) and comparing them to the results of a previous genome scan study based on SNP markers that included whitefish populations of Cliff Lake and Indian Pond (Renaut et al. 2011). Parallel patterns of divergence among species pairs were also considered as signatures of divergent natural selection (Schluter and Nagel 1995).

Because of their recent evolutionary origins (Bernatchez and Dodson 1991), fixed genetic differences between dwarf and normal whitefish are rare and none had been identified in the coding region of malate dehydrogenase prior to this study (Jeukens et al. 2010; Renaut et al. 2010), hence analyses based on both polymorphic and divergent sites among lineages, such as the McDonald–Kreitman test (McDonald and Kreitman 1991), were unlikely to be informative.

### Coding sequence analyses

The Datamonkey server offers tools specifically designed to detect signatures of positive and negative selection from coding sequence alignments based on the nonsynonymous to synonymous substitution rate ratio (d_N_/d_S_). The partitioning approach for robust inference of selection (PARRIS) method was used to detect selection in the alignment as a whole while fixed effects likelihood (FEL), random effects likelihood (REL), and single likelihood ancestor counting (SLAC) methods were applied to detect specific codon sites under positive or negative selection by estimating site-by-site d_N_/d_S_. While these three methods are based on very different approaches, the results they produce are generally in agreement (Kosakovsky Pond and Frost 2005b). Because d_N_/d_S_ for the entire sequence can be smaller than one while specific sites are under positive selection, codon-based approaches are much more powerful for detecting adaptive molecular evolution (Nielsen and Yang 1998).

While a nonsynonymous substitution always causes a change of amino acid in the protein, this change does not necessarily affect the activity of the protein, for instance, through interference with its various binding sites (Ng and Henikoff 2006). We have positioned amino acids corresponding to the identified nonsynonymous substitutions and binding sites of MDH1 on its three-dimensional (3D) structure using PyMOL v.1.3 (DeLano Scientific, Palo Alto, CA). This 3D structure was predicted from the crystal structure of a ternary complex of porcine cytoplasmic malate dehydrogenase (*Sus scrofa*, 78% identity, http://www.rcsb.org/pdb/explore/explore.do?structureId=5MDH) using the 3D-JIGSAW Protein Comparative Modeling Server (v.2.0, http://bmm.cancerresearchuk.org/~3djigsaw/).

## Results

### Sequence processing and annotation

Sequencing results are summarized in Table 1. The trimmed regulatory region was 781-bp long and contained a total of 16 SNPs, whereas the trimmed coding region was 807-bp long and contained 19 SNPs. However, eight coding SNPs were not at HWE and six of these were essentially always heterozygous, hence sequence data for the coding region were likely to be a combination of paralogous sequence variants (PSVs) (Hayes et al. 2007). As a result, haplotypes could not be reconstructed. Five SNPs were shared among all whitefish species, and two were shared among all coregonine species. All of these but one (position 61) were part of the heterozygous SNPs. Except for vendace, which was the most genetically diverse group for the coding region, each population had from zero to three true SNPs.

**Table 1:** *MDH1* regulatory and coding polymorphism within coregonine populations

Continent	Coregonine population^1^	Regulatory SNP positions^2^	Coding SNP positions^3^	Paralogous SNP positions^4^	Nonsynonymous substitutions
North America	Cliff Lake, Normal	373	61, 130, 471, 570, 609, 696	130, 471, 570, 609	130
	Cliff Lake, Dwarf	373	61, 130, 471, 570, 609, 696	130, 471, 570, 609	130
	Indian Pond, Normal	373	61, 130, 471, 570, 609, 696	130, 471, 570, 609	130
	Indian Pond, Dwarf	373	61, 130, 471, 570, 609, 696	130, 471, 570, 609	130
	Cisco	373, 478, 520	130, 471, 489	130, 471, 489	130
Europe	Pasvik River, Benthic	188, 236, 373, 374, 408, 478, 572, 573, 702	61, 130, 471, 570, 609, 634	130, 471, 570, 609	130, 634
	Pasvik River, Limnetic	188, 236, 373, 374, 408, 478, 572, 573, 702	61, 130, 471, 570, 609, 634	130, 471, 570, 609	130, 634
	Lake Zurich, Benthic	236, 373, 374, 408, 491, 572, 573, 702	61, 130, 213, 471, 525, 570, 609	130, 471, 570, 609	130
	Lake Zurich, Limnetic	163, 236, 373, 374, 408, 491, 572, 573, 702	61, 130, 213, 471, 525, 570, 609	130, 471, 570, 609	130
	Vendace	149, 236, 244, 373, 374, 408, 458, 580, 702	39, 103, 108, 112, 130, 150, 213, 378, 429, 471, 570, 582, 602, 609	112, 130, 471, 570, 609	108, 130, 602

1 Cliff Lake and Indian Pond: lake whitefish, Pasvik River catchment and Lake Zurich: European whitefish.

2 Position in the 781-bp regulatory sequence, which corresponds to positions 17,590–18,370 in Genbank accession HQ287747.

3 Position in the 807-bp coding sequence, which corresponds to positions 18,317–24,113 without introns in Genbank accession HQ287747, begins at start codon.

4 Position in the coding sequence of SNPs for which essentially all fish were heterozygous. These SNPs are likely to be sequence differences between paralogous sequence variants.

In contrast to the coding region, none of the SNPs of the regulatory sequence significantly departed from HWE within any of the populations or species analyzed, indicating that they likely represented a single gene copy (Table 1). Haplotypes were successfully reconstructed, with all but five fish having a haplotype combination of confidence probability >0.95. Of the five individuals with probability <0.95, only two remained ambiguous, as none of the possible haplotype combinations had a probability >0.5. Results for the regulatory region in Table 1 also highlight the striking difference in polymorphism rate (SNPs/bp) between American and European populations, the latter group showing much more genetic diversity. In fact, while this rate was relatively similar among populations for the coding region, it was about six times higher in Europe compared to North America for the regulatory region. The unique SNP of North American whitefish was shared among all species.

While annotation for the coding region was already available, detailed annotation of the regulatory regions was carried out and is summarized in Table 2. The only SNP available for North American whitefish in that region (position 373, A/T, Table 1) was located 286-bp upstream of the transcription start site (TSS), and will henceforth be referred to as SNP –286. A recombination breakpoint was identified between this SNP and the TSS. While phylogenetic footprinting showed that SNP –286 was not part of a conserved region among species, database scanning revealed that using the T allele instead of the A allele eliminated part of the putative binding sites (Table S1). Moreover, binding simulation pointed to Foxd3 as the most probable transcription factor binding with the A allele, but not with the T allele (probability threshold = 0.1) in whitefish.

**Table 2:** Summary of whitefish *MDH1* regulatory sequence annotation

Region^1^	Position^2^	Annotation^3^
Untranscribed	1–658	SNP A/T (373)
		Recombination breakpoint (458)
		CpG island (429–658)
		11 putative TFBSs overlapping A allele (373), see Table S1 A allele (373) bound to Foxd3 *P* = 0.42; unbound *P* = 0.20
		Five putative TFBSs overlapping T allele (373), see Table S1 T allele (373) unbound *P* = 0.39; bound to Foxd3 *P* = 0.15
		Majority of conserved regions among species 450–658
Untranslated	659–727	Terminal oligopyrimidine tract (TOP) (659–666)
		Musashi binding element (MBE) (666–672)

1 The 5′ limit of the untranslated region was determined according to salmon *MDH1* complete coding sequence (Genbank accession BT060423) and the 5′ extremity of contig 1009 from RNA sequencing (Jeukens et al. 2010).

2 Position in the 781-bp regulatory sequence, which corresponds to positions 17,590–18,370 in Genbank accession HQ287747.

3 Recombination breakpoint: GARD, Datamonkey (Kosakovsky Pond and Frost 2005a), CpG island: The Sequence Manipulation Suite (Stothard 2000), Putative transcription factor binding sites (TFBSs): JASPAR CORE Vertebrata (Bryne et al. 2008), Posterior probability in binding simulation (*P*): Sunflower (Hoffman and Birney 2010), Conserved regions: ConSite (Sandelin et al. 2004) and the MEME suite (Bailey et al. 2009), untranslated region: UTRsite (Mignone et al. 2005). Positions in the 781-bp regulatory sequence are indicated for each element.

**Figure 2:**
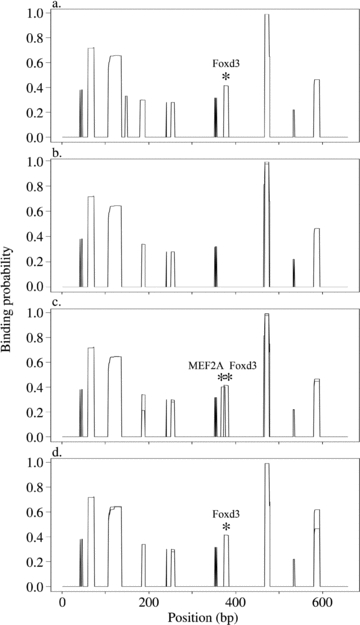
Putative binding profile of *MDH1* regulatory region among coregonine species. Binding probability: highest posterior probability of being bound to a given transcription factor for each of 658-bp upstream of the transcription start site according to binding simulation using Sunflower (Hoffman and Birney 2010), the value of zero was attributed to a given position when the posterior probability of the unbound state was highest. (A) Lake whitefish (*C. clupeaformis*), two haplotypes, (B) lake cisco (*C. artedi*), three haplotypes, (C) European whitefish (*C. lavaretus*), 13 haplotypes, (D) vendace (*C. albula*), four haplotypes. *Putative binding sites that are unbound (probability = 0) for one or more haplotypes depending on alleles at positions 373 and 374 (Table 3), labeled with putative transcription factor name.

Binding simulation was conducted for the full regulatory region upstream of the TSS for all 22 unambiguous haplotypes of this study (Table 3; Fig. 2). Results showed that putative binding profiles were fairly similar among haplotypes and species, but with a few exceptions. First, the plateau that overlaps position 373 (i.e., SNP –286) in Figure 2A corresponds to a putative binding site for Foxd3. All species but the cisco had haplotypes with this binding site, associated with the A allele at position 373. These species also had haplotypes with the T allele at position 373, which lacked this binding site (probability = 0, Fig. 2). This difference between the cisco and the other species was due to a fixed mutation at position 381. Second, the T allele at position 373 combined with the T allele at position 374 in European whitefish was associated with a plateau that extends from positions 367 to 376 in Figure 2C. MEF2A was the most probable transcription factor for this location. A single vendace individual that had an ambiguous genotype was heterozygous at position 374, hence MEF2A may act upon this region in vendace as well.

**Table 3:** Polymorphic positions of 22 unique *MDH1* regulatory haplotypes identified among coregonine species

		Position^2^																		
		
Continent	Haplotype^1^	149	152	163	188	197	236	373	374	381	408	458	460	478	491	520	572	573	580	643
North America	Cocl 1	T	A	G	G	C	A	A	A	G	G	A	A	C	C	C	C	C	A	G
	Cocl 2	.	.	.	.	.	.	T	.	.	.	.	.	.	.	.	.	.	.	.
	Coar 1	.	C	.	.	A	.	T	.	T	.	.	C	.	.	T	.	.	.	A
	Coar 2	.	C	.	.	A	.	T	.	T	.	.	C	.	.	.	.	.	.	A
	Coar 3	.	C	.	.	A	.	.	.	T	.	.	C	T	.	.	.	.	.	A
Europe	Cola 1	.	C	.	.	A	C	.	.	.	T	.	C	.	.	.	.	.	.	A
	Cola 2	.	C	.	.	A	C	T	.	.	T	.	C	.	.	.	.	.	.	A
	Cola 3	.	C	.	.	A	C	T	.	.	T	.	C	T	.	.	.	.	.	A
	Cola 4	.	C	.	.	A	.	T	T	.	T	.	C	.	.	.	.	.	.	A
	Cola 5	.	C	.	.	A	.	T	T	.	.	.	C	.	.	.	T	G	.	A
	Cola 6	.	C	.	A	A	.	T	T	.	.	.	C	.	.	.	.	.	.	A
	Cola 7	.	C	.	.	A	C	.	.	.	T	.	C	.	.	.	T	G	.	A
	Cola 8	.	C	.	.	A	C	.	.	.	T	.	C	.	.	.	.	.	.	A
	Cola 9	.	C	.	.	A	.	.	.	.	.	.	C	.	T	.	T	G	.	A
	Cola 10	.	C	.	.	A	.	.	.	.	.	.	C	.	.	.	T	G	.	A
	Cola 11	.	C	.	.	A	.	T	T	.	.	.	C	.	.	.	.	.	.	A
	Cola 12	.	C	T	.	A	.	T	T	.	.	.	C	.	.	.	.	.	.	A
	Cola 13	.	C	.	.	A	.	T	.	.	.	.	C	.	.	.	T	G	.	A
	Coal 1	.	C	.	.	A	C	T	.	.	T	.	C	.	.	.	.	.	G	A
	Coal 2	.	C	.	.	A	C	T	.	.	T	.	C	.	.	.	.	.	.	A
	Coal 3	.	C	.	.	A	.	T	.	.	.	.	C	.	.	.	.	.	G	A
	Coal 4	C	C	.	.	A	.	.	.	.	.	C	C	.	.	.	.	.	G	A

1 Cocl = lake whitefish (*C. clupeaformis*), Coar = lake cisco (*C. artedi*), Cola = European whitefish (*C. lavaretus*), Coal = vendace (*C. albula*).

2 Position in the 658-bp upstream of the transcription start site (positions 17,590–18,247 in Genbank accession HQ287747).

### MDH1 regulatory region

**Figure 3:**
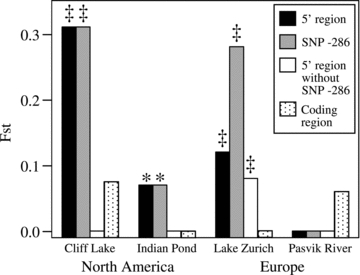
Genetic differentiation of *MDH1* 5′ regulatory and coding regions between North American and European whitefish species pairs. Based on 781 bp of regulatory sequence (positions 17,590–18,370 in Genbank accession HQ287747) and 807 bp of coding sequence (positions 18,317–24,113 without introns in Genbank accession HQ287747). *F*_ST_ values based on overall mean pairwise genetic p-distances computed with HYPHY (Kosakovsky Pond et al. 2005). Negative *F*_ST_ estimates were forced to zero. For the 5′ regulatory region, both alleles for each fish were used. For the coding region, mean *F*_ST_ for true single nucleotide polymorphisms (SNPs) (excluding paralogous SNPs, see Table 1) was computed by using one copy of each observed haplotype per fish. ‡ Bootstrapped estimator significantly different from zero (*P* < 0.05, 500 replicates) and probability of a random *F*_ST_ greater than the observed value <0.05 (500 permutations). *Probability of a random *F*_ST_ greater than the observed value <0.05 (500 permutations).

SNP –286 showed divergent allele frequencies in three of the four independently evolved whitefish species pairs of this study (Fig. 3). *F*_ST_ values for this specific position were consistently higher than those for the rest of the regulatory sequence in these pairs. They were also higher than *F*_ST_ values for the coding region, although these estimates were more conservative due to the use of a single copy per haplotype, per individual (i.e., one haplotype for homozygotes and two for heterozygotes). Moreover, the T allele at SNP –286 was more frequent in limnetic fish in all three cases of divergence, with frequencies of 0.67 in Cliff Lake versus 0.2 for normals, 0.3 in Indian Pond versus 0.1 for normals, and 0.73 in Lake Zurich versus 0.32 for the benthic population. This allele was also more frequent in the limnetic vendace and cisco as well as in the Pasvik catchment, with frequencies of 0.63, 0.67, and 0.67, respectively. *F*_ST_ values at SNP –286 for Cliff Lake and Indian Pond were comparable to mean *F*_ST_ values from a genome scan using 94 coding SNP markers (0.28 for Cliff, 0.06 for Indian, Renaut et al. 2011).

Evolution of the regulatory sequence was modeled separately on each side of the identified recombination breakpoint. The HKY85 substitution model, which allows for unequal base frequencies and unequal transversion and transition rates (Hasegawa et al. 1985), was selected for model fitting with global parameters. This means that there is one transition rate (α) and one transversion rate (β) for all branches of the phylogenetic tree. Using local branch parameters did not improve the model, with a difference of only 11 units of likelihood score for 88 additional parameters. Global model fitting showed that κ, the transversion/transition rate ratio (β/α), was equal to 2.93 upstream and 0.41 downstream of the recombination breakpoint. An LRT using constrained model κ_upstream_ = κ_downstream_ as the null hypothesis confirmed the significance of this difference (*P*-value = 0.02, parametric bootstrap, 100 replicates).

### MDH1 coding region

The SLAC method allows for ambiguous reconstructions of ancestral codons by averaging over all possible codon states (Kosakovsky Pond and Frost 2005b). It is therefore well suited when sequence ambiguities are assumed to represent polymorphism, as was the case for our coding sequence dataset that appeared to be a mixture of gene copies. SLAC analysis of the 24 unique coding haplotypes identified in this study revealed that the *MDH1* coding sequence was under purifying selection among coregonine species, with mean codon-specific d_N_/d_S_ = 1.73e^–15^. Other tools of the Datamonkey server (PARRIS, FEL, and REL) also pointed to strong purifying selection, although their treatment of ambiguities was slightly different (results not shown).

**Figure 4:**
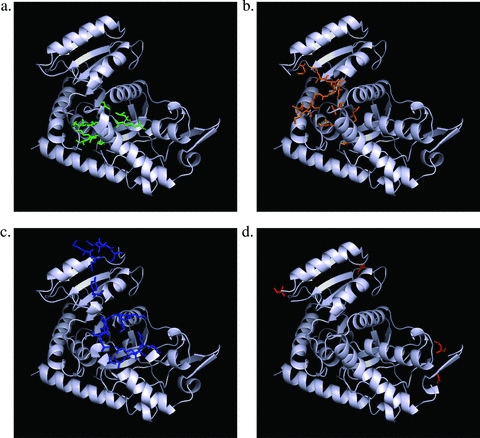
Predicted ternary structure of whitefish MDH1. Based on whitefish MDH1 protein sequence (Genbank accession ADV02378) of 78% homology with porcine cytoplasmic malate dehydrogenase in the Protein Data Bank (ID = 5MDH) using the 3D-JIGSAW server (v.2.0, http://bmm.cancerresearchuk.org/~3djigsaw/). The graphical representation was created with Pymol v.1.3. (A) Green = amino acid residues of the malate binding domain, (B) orange = amino acid residues of the NAD binding domain, (C) blue = amino acid residues of the dimer interface, (D) amino acid changes due to nonsynonymous substitutions among coregonine populations (see Table 1).

The 3D structure of whitefish MDH1 was successfully predicted from porcine MDH1. In addition to the three binding domains, that is, malate and NAD binding as well as dimer interface, the four amino acids associated with nonsynonymous substitutions within coregonine populations were positioned on this predicted structure (Fig. 4). Results showed that these changes were somewhat peripheral in the ternary structure of the protein. Moreover, they did not fall within or close to the three binding domains.

## Discussion

### Expression of PSVs in whitefish

The study of salmonid genomes is particularly challenging due to their pseudotetraploidy (Allendorf and Thorgaard 1984), which translates into the occurrence of recently diverged PSVs (Hayes et al. 2007; Moen et al. 2008). According to model fitting in Atlantic salmon, the average SNP density (SNPs/bp) in duplicated regions of the genome was approximately three times that of unduplicated regions. This is consistent with *MDH1* in dwarf and normal whitefish, where only two of the six coding SNPs likely corresponded to a single gene copy (probably one per PSV, results not shown). Isozyme studies have demonstrated that the salmonid MDH1 homodimer forms from four different subunits, A, A’, B, and B’, the A type being predominant in liver tissue (Bailey et al. 1970; Allendorf et al. 1977). Since A- and B-type subunits markedly differ in amino acid composition (Bailey et al. 1970; Wheat et al. 1972), whitefish *MDH1* PSVs in this study, which differ by only one amino acid, probably encode the A and A’ subunits. Once a reference genome becomes available for salmonid fishes, it should be possible to delineate the evolutionary history of gene families such as *MDH1*.

Some of the coding SNPs presented in this study showed pronounced allele frequency differences between sympatric normal and dwarf whitefish in a previous RNA sequencing experiment (Renaut et al. 2010). One of them (position 570, Table 1) clearly was a sequence difference between PSVs rather than a true SNP. As allele frequencies deduced from RNA sequencing are representative of allele-specific expression levels (e.g., Jeukens et al. 2010), this strongly suggests that overexpression of *MDH1* in dwarf whitefish involves divergence in PSV expression levels. This phenomenon would deserve further investigation, particularly considering that gene duplication appears to promote regulatory evolution (Gu et al. 2005; Dong et al. 2011). Hence, PSV expression divergence might play an important role in transcriptomic divergence among whitefish species pairs as well as in salmonid fishes in general.

### Purifying selection acting upon MDH1 coding region

Because *MDH1* coding sequence data were a combination of PSVs, haplotypes could not be reconstructed. However, this appeared to be the case for all coregonine populations included in the study, as they all deviated from HWE for part of their SNPs, two of which were common to all species (positions 130 and 471, Table 1). There was also evidence that both PSVs are actually exploited by liver tissue cells, as this mixture of gene copies was amplified from cDNA. Data were therefore analyzed together, as was previously done for bulk viral mixtures, where each sequence represented a unique patient (Poon et al. 2007; Kosakovsky Pond et al. 2009). Results from likelihood-based analyses of sequence evolution pointed toward purifying selection acting upon the *MDH1* coding mixture. Relative positions of the four amino acid changes within the ternary protein structure were also consistent with purifying selection, as they all seemed unrelated to those of MDH1 binding domains. Although this remains a visual inference, these peripheral changes are unlikely to interfere with protein activity. Given that the coding sequence for this mixture of PSVs is clearly under purifying selective pressures among coregonine fishes, analysis of a single *MDH1* gene copy would very likely have led to the same conclusion.

### Regulatory evolution of MDH1

Generally speaking, promoters are located upstream and relatively close to the genes they regulate (White 2001). The core promoter, which normally extends a few tens of base pairs upstream of the TSS, contains general TFBSs that are necessary to initiate transcription and was part of the most conserved region among species for *MDH1*. The proximal promoter, which usually extends a few hundreds of base pairs upstream of the TSS and contains specific TFBSs, was also relatively conserved up until position ∼450, close to the putative recombination breakpoint. SNP –286 is also likely to be part of the *MDH1* proximal promoter, but upstream of this breakpoint. According to binding simulation, the A allele at SNP –286, which was most common in normal whitefish and benthic European whitefish from Lake Zurich, most likely binds Foxd3, while the T allele, which was most common in dwarf North American whitefish and limnetic European whitefish, is more likely to be unbound. Foxd3, or forkhead box D3, is conserved in human, chimpanzee, dog, cow, mouse, and zebrafish (NCBI Gene ID: 29203). Members of the Fox gene family are implicated in a wide range of biological processes, including hepatic glucose metabolism and energy metabolism (Le lay and Kaestner 2010). Both positive and negative regulation of transcription have been associated with this transcription factor, hence it could cause negative regulation in whitefish *MDH1*, as this gene is underexpressed in normal whitefish liver, where the allele that potentially binds Foxd3 occurs more frequently. Binding simulation also revealed that European whitefish, due to a second SNP located immediately downstream of SNP –286, possibly had another binding site. In fact, the T allele at this second SNP, which is also most common in limnetic fish from Lake Zurich, introduced a putative binding site for MEF2A, or MADS box transcription enhancer factor 2, polypeptide A. This transcription factor is conserved in chimpanzee, dog, cow, mouse, chicken, zebrafish, fruit fly, and mosquito and normally activates many muscle-specific, growth factor induced, and stress-induced genes (NCBI Gene ID: 4205). Therefore, European whitefish, depending on their haplotypes for these two adjacent SNPs, might have three potential binding statuses: unbound by TA, bound to Foxd3 by AA, and bound to MEF2A by TT. However, the AT haplotype was never observed in this study. Of course, until functional validation is performed, linkage disequilibrium between these two SNPs and the actual cause of *MDH1* expression difference as well as differences in other regulatory components (e.g., transcription factors or enhancers further upstream) cannot be ruled out, given the complexity of eukaryotic promoters.

**Figure 5:**
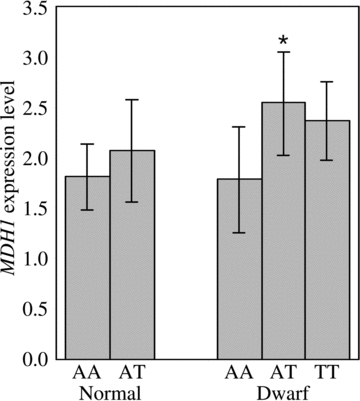
*MDH1* expression level as a function of the genotype at SNP –286 in dwarf and normal lake whitefish. Expression level: normalized R/Lowess signal intensity in log_2_ from a previous microarray experiment (St-Cyr et al. 2008). Data for 16 fish from Cliff Lake and 15 from Indian Pond, half normals (N), half dwarves (D). Frequency of the T allele: Cliff N = 0.2, D = 0.67 and Indian N = 0.1, D = 0.3. One-way ANOVA between the five groups: *P*-value = 0.007. *Tukey multiple comparisons of means: Dwarf AT/Dwarf AA *P*-value = 0.05, Dwarf AT/Normal AA: *P*-value = 0.007.

A previous whitefish genome scan study showed that SNP markers from candidate loci associated with adaptive phenotypes on the basis of gene expression differences did not show reduced gene flow (outlier *F*_ST_ values) compared to all other markers (Renaut et al. 2011). This is consistent with *F*_ST_ values computed for SNP –286, hence these values provide no direct evidence of divergent natural selection. However, parallelism in genetic differentiation at this SNP among three independent whitefish species pairs from two continents, two of which did not emerge following secondary contact (Douglas et al. 1999; Lu et al. 2001), is very unlikely to have evolved by random processes (Schluter and Nagel 1995). Moreover, there appears to be an association between genotype at this SNP and *MDH1* expression level in North American whitefish species (Fig. 5). Altogether, these results provide evidence for the role of natural selection acting on regulatory regions responsible for *MDH1* expression divergence among whitefish species pairs.

### Coding versus regulatory evolution

While *cis*-regulatory changes affect transcription in a gene-specific manner (e.g., TFBS), *trans*-regulatory changes modify factors that interact with *cis*-regulatory elements of one or multiple genes (Davidson 2001). *Cis*-acting changes in TFBSs might underlie the evolution of gene expression divergence in whitefish. However, as gene expression and coding sequence divergence do not seem to have acted upon the same genes during whitefish evolution (Jeukens et al. 2010), this would be possible only if recombination has decoupled regulatory and coding regions of genes (Kohn et al. 2008). Results for *MDH1* suggest that this premise is realistic, as a putative recombination breakpoint was identified 200-bp upstream of the TSS. Of course, *trans*-regulation of genes is likely implicated as well, especially considering the previous identification in whitefish of genomic regions with pleiotropic effects on gene expression (Derome et al. 2008; Whiteley et al. 2008).

DNA substitutions are of two kinds: transitions, that is, interchanges of two purines (A↔G) or two pyrimidines (C↔T), and transversions, that is, interchanges of purines and pyrimidines. Transversions are therefore associated with structural changes in DNA molecules. While there are twice as many possible transversions, transition mutations are generally more frequent, especially in protein sequences, where they are less likely to cause a change of amino acid (Wakeley 1996). However, this is not always the case. For instance, in grasshopper pseudogenes, the transversion/transition rate ratio was equal to 1, hence it was consistent with neutral expectations (Keller et al. 2007). Here, in the 5′ regulatory region of *MDH1* upstream of the recombination breakpoint, this ratio was almost three times higher than expected. Conversely, the transversion/transition ratio downstream of the TSS was more consistent with the widespread transition bias. This result might reflect relaxation of purifying selective pressures and/or diversifying selection in the proximal and distal promoter of *MDH1*, in opposition to the purifying selection acting upon its coding region.

### Standing genetic variation in regulatory regions among whitefish species pairs

During Pleistocene glaciations, North American ice sheets were particularly large, forcing freshwater fish to survive in fringe habitats and proglacial lakes formed by meltwater along glacial margins (Dyke and Prest 1987). Habitat loss caused by glacial advances and survival in these restricted glacial refugia have caused substantial loss of genetic diversity (Avise et al. 1984). Thus, fish species from glaciated regions exhibit lower intraspecific diversity compared to species from nonglaciated areas (Bernatchez and Wilson 1998). Glaciation effects are particularly obvious in whitefish whereby North American populations are characterized by much lower levels of genetic diversity relative to European populations, in accordance with the much smaller extent of Eurasian ice sheets (Bernatchez and Dodson 1994). Results presented here are consistent with this general pattern, as all European whitefish populations as well as the vendace showed higher levels of polymorphism in the regulatory region compared to the North American lake whitefish and lake cisco. It is also noteworthy that, in addition to their depleted genetic diversity, sympatric pairs of the North American lake whitefish show reduced phenotypic diversity relative to European populations. Thus, sympatric pairs of European whitefish show higher levels of phenotypic differentiation between limnetic and benthic fish (e.g., difference in mean gill-raker number of two in North America, Lu and Bernatchez 1999; and 12 in Norway, Østbye et al. 2006). Moreover, more than two sympatric forms have regularly emerged following glacial retreat in European lakes (e.g., 11 populations in Lake Femund, Norway, Østbye et al. 2005). This raises the hypothesis that the extent of genetic polymorphism in regulatory regions may have fuelled divergent selection toward varying degrees of phenotypic differentiation between North American and European whitefish species pairs (e.g., Renaut et al. 2011).

According to binding simulation for 22 regulatory haplotypes of *MDH1*, putative binding profiles were fairly conserved among species, despite sequence variation for 16 intraspecific SNPs distributed along most of the regulatory region. This is consistent with the observation that strong stabilizing selection generally maintains expression patterns despite rapid promoter evolution (Denver et al. 2005; Tirosh et al. 2008). The only true exception to this rule in our data was the small region around SNP –286, further supporting a regulatory role for this SNP, which almost certainly represents standing genetic variation as it was polymorphic in all coregonine species of this study. While SNP –286 was most probably unbound in all lake cisco haplotypes, European populations had an additional putative binding state compared to North American whitefish due to an SNP variant at position –285. As this SNP was shared among European whitefish and the distantly related vendace, it is also likely to represent standing genetic variation. Hence, in genes such as *MDH1* for which the protein sequence appears to evolve under strong purifying selection, standing genetic diversity in the regulatory region may have contributed more to adaptive divergence than the coding region through changes in gene expression levels. Clearly, the relation between regulatory standing genetic variation and phenotypic diversity among sympatric pairs of whitefish from North America and Europe would deserve further investigation, especially considering that standing variation has great potential to facilitate rapid adaptation to new environments (reviewed by Barrett and Schluter 2007).

## Conclusion

The main goal of this study was to identify signatures of natural selection in the coding and regulatory sequences of a candidate gene thought to be implicated in adaptive metabolic divergence among whitefish species pairs. Results obtained for *MDH1* showed that, while purifying selection is preserving the integrity of the *MDH1* protein, an SNP at position –286 in the proximal promoter region exhibits parallel allele frequency divergence among independent sympatric pairs of whitefish from North America and Europe. Moreover, there appears to be an association between genotype at this SNP and *MDH1* expression level. These results provide evidence for the role of divergent natural selection in the regulatory evolution of this gene among whitefish species pairs. Moreover, they bring support to the hypothesis that the level of standing genetic variation influences the potential for adaptive phenotypic divergence. Further sequencing efforts in whitefish (e.g., Jeukens et al. 2011) and the completion of whole genome sequence in other salmonids (e.g., Atlantic salmon) combined with technological progress should enrich our knowledge of the whitefish genome and contribute to a more comprehensive understanding of the mechanisms and relative importance of regulatory and coding sequence evolution in ongoing speciation events.

## Data Accessibility

*MDH1* DNA sequence: Genbank accession HQ287747.

MDH1 protein sequence: Genbank accession ADV02378.

## Supporting Information

**Table S1.** Putative transcription factor binding sites(TFBSs) overlapping SNP –286 of the *MDH1* gene.
