# Supplementary material for: Regulatory versus coding signatures of natural selection in a candidate gene involved in the adaptive divergence of whitefish species pairs (Coregonus spp.)
Source: Ecol Evol. 2012 Jan;2(1):258–71. doi: 10.1002/ece3.52 (PMC3297193; doi:10.1002/ece3.52)
Supplement: Supplementary file 1 [file ece30002-0258-SD1.pdf]

Table S1 Putative transcription factor binding sites (TFBSs) overlapping SNP -286 of the *MDH1* gene

From a scan of the JASPAR CORE Vertebrata database (http://jaspar.genereg.net/) with the A allele at SNP -286.
Each TFBS in the database has a profile model known as a position-specific weight matrix.
Model ID: unique identifier for each model, annotations available in the JASPAR database
Model name: The name of the transcription factor.
Score: based on the match of the sequence to the matrix model
Relative score: percentage of the best possible score for a given TFBS model, threshold of 80%.
Predicted site sequence: capital letters indicate the positions in the sequence which match with the core sequence of the matrix
Not identified with T allele: no match to this model when the T allele is in the sequence instead of the A allele.
A total of 5 TFBS models overlapped the T allele: ARID3A, FOXL1 and CEBPA on the minus strand, and ARID3A and SRY on the plus strand

Model ID	Model name	Score	Relative score	Start	End	Strand	Predicted site sequence	Not identified with T allele
MA0041.1	Foxd3	10.594	0.881	373	384	minus	GTACATTTTTTt	√
MA0151.1	ARID3A	5.399	0.826	370	375	minus	TTtAAA	
MA0102.2	CEBPA	6.246	0.821	369	377	minus	TTTTtAAAT	
MA0033.1	FOXL1	7.158	0.929	368	375	minus	TTtAAATA	
MA0151.1	ARID3A	5.399	0.826	369	374	plus	ATTTaA	
MA0151.1	ARID3A	5.399	0.826	370	375	plus	TTTaAA	
MA0084.1	SRY	5.725	0.801	371	379	plus	TTaAAAAAA	
MA0108.2	TBP	6.287	0.812	369	383	plus	ATTTaAAAAAATGTA	√
MA0084.1	SRY	6.394	0.824	372	380	plus	TaAAAAAAT	
MA0124.1	NKX3-1	6.555	0.819	367	373	plus	TTATTTa	√
MA0052.1	MEF2A	7.917	0.810	367	376	plus	TTATTTaAAA	√
